# Assessment of Satisfaction Levels Among Families of Intensive Care Unit Patients in Saudi Arabia: A Cross-Sectional Study

**DOI:** 10.1155/2024/8481083

**Published:** 2024-10-23

**Authors:** Abdullah Shbeer, Mohammed Ageel

**Affiliations:** Department of Surgery, College of Medicine, Jazan University, Jazan, Saudi Arabia

**Keywords:** communication, decision-making, family satisfaction, intensive care unit, quality improvement

## Abstract

**Background:** Regularly measuring family satisfaction with intensive care unit (ICU) experience is crucial for ensuring high-quality care and identifying areas for improvement. This study aimed to evaluate family satisfaction with the ICU in Saudi Arabia.

**Methods:** A cross-sectional survey was conducted among 248 family members of patients admitted to various ICUs. The survey assessed family satisfaction via a validated questionnaire, the Critical Care Family Satisfaction Survey (CCFSS), which includes five subscales: assurance, information, comfort, proximity, and support. Demographic data were also collected. Descriptive and inferential statistics were calculated.

**Results:** The demographic distribution revealed that a majority of the participants were female (70.97%, *n* = 176), with the relationships with the patients predominantly being parents (41.94%, *n* = 104) or offspring (33.87%, *n* = 84). The overall satisfaction score was 3.79 ± 1.26, with 66.13% of the participants reporting high satisfaction, 20.97% reporting intermediate satisfaction, and 12.90% reporting low satisfaction. The mean subscale scores were as follows: assurance (3.82 ± 1.2), information (3.83 ± 1.25), comfort (3.81 ± 1.27), proximity (3.72 ± 1.28), and support (3.78 ± 1.28). The highest satisfaction scores were observed for sharing in decisions, noise levels, and staff honesty, whereas the lowest scores were for visiting hours flexibility, transfer preparation, and staff responsiveness. Males reported significantly greater satisfaction (4.24 ± 1.20) than females did (3.61 ± 1.11, *p* = 0.007).

**Conclusions:** This study revealed moderate to high levels of family satisfaction with the ICU, with significant differences based on sex. The findings highlight the importance of effective communication, family involvement in decision-making, and supportive ICU policies. ICUs should regularly assess family satisfaction and use the results to guide quality improvement efforts, with a focus on areas with lower satisfaction scores.

## 1. Introduction

In the contemporary healthcare landscape, the intensive care unit (ICU) represents a critical juncture in the provision of medical care for patients facing life-threatening conditions. The ICU experience, characterized by highly technical and specialized care, often entails significant emotional and psychological challenges for both patients and their families [1, 2]. Given the complex nature of ICU care, understanding the perspectives of patients' families is crucial for enhancing the quality of care and ensuring a supportive environment. This understanding extends beyond clinical outcomes, with a focus on the overall satisfaction of families with the ICU stay [3, 4].

Family satisfaction has emerged as a crucial metric for assessing the quality of care in ICUs. The psychological distress and burden borne by families of critically ill patients are substantial, prompting extensive research into their psychological needs [5]. A systematic review identified 27 different questionnaires, with only a few achieving high levels of validity and reliability, such as the Critical Care Family Needs Inventory and the Society of Critical Care Medicine Family Needs Assessment [6]. These studies emphasize the importance of communication, emotional support, and involvement in decision-making as key factors influencing family satisfaction [7]. Other studies have consistently demonstrated that these family members prioritize receiving prompt and clear communication, maintaining proximity to the patient, and establishing a trusting, compassionate understanding with healthcare providers. Such needs have been documented in seminal previous studies [8–10]. The degree of family satisfaction with the care provided is believed to correlate directly with how well these psychological and informational needs are met, alongside the expectations regarding the care extended to both them and the patient during hospitalization [11]. The growing focus on family satisfaction has led to the formulation of clinical practice guidelines and consensus statements, which advocate addressing the needs of family members during critical illness as a surrogate marker for the quality of patient-centered critical care [11–13].

Studies focusing on patient and family satisfaction are pivotal for the continuous improvement of healthcare services, as they provide direct feedback on the care received and highlight areas needing enhancement. In the context of Saudi Arabia, where substantial progress in healthcare has been made over the last decade, understanding and managing patient and family satisfaction has become even more crucial. Despite the importance of such indicators for hospital performance, there remains a scarcity of documented research on the satisfaction of ICU patients' families as an indirect indicator of patient satisfaction and how this satisfaction is managed within the country. The aim of this study was to explore the levels of satisfaction among families of ICU patients in Saudi Arabia.

## 2. Materials and Methods

### 2.1. Study Design and Setting

A cross-sectional study design was carried out from January 2023 to January 2024. The study was conducted across multiple ICUs within the Jazan region in Saudi Arabia.

### 2.2. Study Population and Sample Size

The study targeted family members of patients who had been admitted to the ICU. Participants were recruited by reaching out to family members of patients who had been in the ICU for more than 72 h. The inclusion criteria specified that participants must be 18 years or older and have a patient who had been cared for in an ICU for at least 24 h. To calculate the sample size for a study with an effect size of 0.50, a significance level of 0.05, and a power of 0.95, we utilize a power analysis method for comparing means between two independent groups. Key parameters include the medium effect size (*d* = 0.50), the two-tailed significance level (*α* = 0.05) with its associated critical value *Z*_1−*α*/2_ of approximately 1.96, and the power (1 − *β* = 0.95) with a critical value for *Z*_1−*β*_. The sample size per group is calculated using the following formula:

(1) $$\begin{array}{c} n=2\;{\left(\frac{\left(\;Z_{1-\alpha /2}\right)+\left(\;Z_{1-\beta }\right)}{d}\right)}^{2}. \end{array}$$

The calculation determined that approximately 104 participants are needed per group, leading to a total sample size of about 208 participants for a study with two groups.

### 2.3. Recruitment and Sampling

In this study, a convenience sampling method was employed to recruit participants. The recruitment process involved identifying eligible participants through the ICU care team, who played a crucial role in facilitating recruitment by introducing the research team to potential participants and assisting with informed consent. Among the 300 family members of ICU patients contacted by data collectors, 248 responded to the survey, resulting in a response rate of 82.67%.

### 2.4. Ethical Considerations

Ethical approval for this study was obtained from the Institutional Review Board at Jazan University (Approval no. 43/C/1773). Written informed consent was obtained from all participants prior to data collection. The consent process and documentation were formally approved by the ethics committee.

### 2.5. Data Collection

The first section of the survey collected the sociodemographic characteristics of the participants, including sex, relationship to the patient, and type of ICU. The second section used the previously validated Arabic version of the Critical Care Family Satisfaction Survey (CCFSS) [14]. The CCFSS measures family satisfaction, and needs in the critical care setting serve as an indirect indicator of patient satisfaction, evaluating the quality of care in the ICU on the basis of the satisfaction of family members' psychological and informational needs. The CCFSS includes 20 statements, which the respondent is asked to grade on a five-point Likert scale according to their satisfaction with that item. The CCFSS is divided into five subscales: comfort (2 items), proximity (3 items), information (4 items), assurance (5 items), and support (6 items). These subscales reflect distinct dimensions of family satisfaction: assurance focuses on the family's need for hope and reassurance; proximity addresses the need for physical and emotional closeness; information highlights the importance of clear and timely communication; support addresses the necessity for emotional and logistical support; and comfort relates to the physical comfort of family members within the ICU environment. Each item is ranked by the family member on a five-point Likert scale (1 = very much dissatisfied and 5 = very much satisfied).

A mean score is calculated for each subscale and the overall family satisfaction score (ranging from 1 to 5). The primary outcome of the study is the overall satisfaction score, which comprises the five subscales. Higher scores indicate greater satisfaction as perceived by a patient's family member. The satisfaction level is segmented into three levels on the basis of an individual's overall mean score: “low satisfaction” (scores from 1.00 to 2.33), “intermediate satisfaction” (scores from 2.34 to 3.66), and “high satisfaction” (scores from 3.67 to 5.00).

### 2.6. Data Analysis

The Statistical Package for Social Sciences (SPSS 27) was used for data analysis. Descriptive statistics, including means, standard deviations, frequencies, and percentages, were calculated for all the variables. The Mann‒Whitney *U* test (U) and the Kruskal‒Wallis test were used to examine whether there were any differences between the overall mean family satisfaction score and the family's demographic variables. A *p* value < 0.05 was regarded as statistically significant.

## 3. Results

A total of 248 participants were included in the study. The demographic and clinical characteristics of the intensive care patients and their family members are presented in Table 1, along with the overall mean satisfaction scores. Most participants were female (70.97%, *n* = 176), with a mean satisfaction score of 3.61 ± 1.11, which was significantly lower than that of males (4.24 ± 1.20, *p* = 0.007). Most of the participants were parents (41.94%, *n* = 104) or offspring (33.87%, *n* = 84), with no significant differences in the mean satisfaction scores across relationships (*p* = 0.594). Patients were admitted to medical ICUs (50.00%, *n* = 124), pediatric ICUs (24.19%, *n* = 60), and surgical ICUs (25.81%, *n* = 64), with similar satisfaction levels across unit types (*p* = 0.636).

The results of the CCFSS items across various aspects of care in an ICU are presented in Table 2. The results highlight the items with the highest and lowest satisfaction scores. The top-scoring items were sharing in decisions regarding the family member's recovery (3.95 ± 1.11), noise in the ICU (3.92 ± 1.13), and honesty of staff about the family member's condition (3.9 ± 1.17). The lowest scores were for flexibility of visiting hours (3.58 ± 1.31), preparation for transfer from critical care (3.63 ± 1.33), and promptness of staff response (3.71 ± 1.3). Clear communication about tests, procedures, and treatments and the ability to share in care were highly satisfactory, indicating effective engagement. However, lower scores for visiting hours and staff promptness suggest areas for improvement.

Assessing family satisfaction in ICU settings across five subscales—assurance, proximity, information, support, and comfort—the overall findings demonstrated varying levels of satisfaction.  Table 3 shows the subscale mean scores across the five subscales. The mean scores for the subscales were as follows: assurance (3.82 ± 1.20), information (3.83 ± 1.25), comfort (3.81 ± 1.27), proximity (3.72 ± 1.28), and support (3.78 ± 1.28). The overall satisfaction score, which was the primary outcome, was 3.79 ± 1.26.

**Figure 1:**
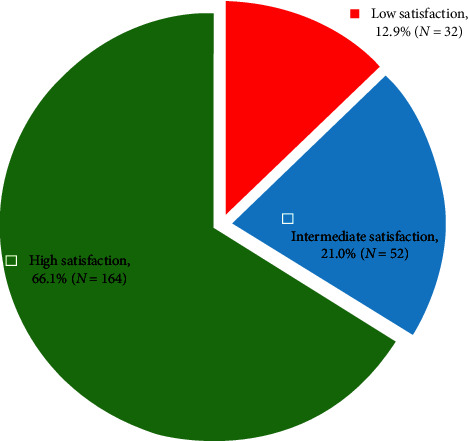
Percentage distribution of the family's level of satisfaction.

Figure 1 shows the percentage distribution of family satisfaction levels, categorized as low (1.00–2.33), intermediate (2.34–3.66), and high (3.67–5.00) on the basis of the scoring system. The majority of the participants (66.13%) reported high satisfaction, 20.97% reported intermediate satisfaction, and 12.90% reported low satisfaction.

## 4. Discussion

Regularly measuring family satisfaction with the ICU is crucial for ensuring high-quality care and identifying areas for improvement. Family satisfaction is a key indicator of the overall quality of care provided in the ICU, as it reflects families' perceptions of the care their loved one receives and the support they provide during challenging times [5]. Assessing family satisfaction can help ICUs identify strengths and weaknesses in their care delivery, communication, and support systems, allowing them to make targeted improvements [15]. The aim of this study was to evaluate family satisfaction with ICU care provision.

The overall satisfaction score in this study was 3.79 ± 1.26, indicating a moderate to high level of satisfaction among the participants. Most of the participants (66.13%) reported high satisfaction, 20.97% reported intermediate satisfaction, and 12.90% reported low satisfaction. Previous studies have reported high levels of family satisfaction with ICUs in general [16–18]. On the other hand, some studies reported low satisfaction levels [1]. The presence of intermediate and low satisfaction levels suggests that there is still room for improvement in certain aspects of ICU care and family support. The slightly lower satisfaction levels in this study than in some previous reports may be due to differences in ICU settings, patient populations, or cultural factors [2, 14, 19].

The mean scores for the subscales in this study were as follows: assurance (3.82 ± 1.2), information (3.83 ± 1.25), comfort (3.81 ± 1.27), proximity (3.72 ± 1.28), and support (3.78 ± 1.28). These findings suggest that families were generally satisfied with the level of assurance, information, comfort, proximity, and support provided by the ICU staff. Compared with a study from 2014 in Saudi Arabia, the current satisfaction scores across all dimensions were higher: assurance (2.39 ± 0.51), information (2.31 ± 0.48), proximity (2.4 ± 0.6), support (2.5 ± 0.5), and comfort (2.5 ± 0.7) [1]. The individual item scores presented in Table 2 provide further insight into specific areas of strength and weakness in ICU family satisfaction. The highest satisfaction scores were observed for sharing in decisions regarding the family member's recovery, noise in the ICU, and honesty of the staff about the family member's condition. These findings highlight the importance of effective communication, transparency, and family involvement in decision-making [20]. The lowest satisfaction scores were reported for flexibility of visiting hours, preparation for transfer from critical care, and promptness of staff response. These results suggest a need for improvement in ICU policies related to visitation, discharge planning, and staff responsiveness [21–23].

The respondent demographics revealed significant differences in satisfaction levels based on gender, with males reporting higher satisfaction (4.24 ± 1.20) than females did (3.61 ± 1.11). This finding is consistent with those of several previous studies that reported gender differences in ICU family satisfaction [21, 23]. The reasons for these gender differences are not entirely clear but may be related to differences in communication styles, emotional needs, or expectations [21]. Further research is needed to better understand and address these gender disparities in ICU family satisfaction.

The implications of this study are that ICUs should regularly assess family satisfaction and use the findings to guide quality improvement efforts. Particular attention should be given to areas with lower satisfaction scores, such as visiting hours, discharge preparation, and staff responsiveness. ICUs should also consider strategies to address gender differences in satisfaction, such as providing targeted support and communication for female family members. This study provides a foundation for future research aimed at improving family experiences in ICU settings. A promising avenue for further investigation involves examining the effects of targeted interventions designed to enhance family satisfaction during ICU experiences. Future research could systematically assess interventions such as improved communication strategies, family-centered care models, and structured support programs to evaluate their effectiveness in increasing overall family satisfaction. This, in turn, serves as an indirect measure of patient satisfaction and the quality of care provided in ICU environments.

### 4.1. Limitations

This study has several limitations that should be considered when interpreting the findings. First, the cross-sectional design and convenience sampling method may limit the generalizability of the results to broader populations. Although the response rate was relatively high at 82.67%, the potential for nonresponse bias existed because 52 family members did not participate. The characteristics of these nonrespondents are unknown, and it is possible that dissatisfied family members may have opted not to participate, potentially skewing the results toward more favorable evaluations of the ICU. In addition, the study was conducted in a multicenter setting within a single regional healthcare system, which may limit the applicability of the findings to other regions or healthcare settings. The use of self-reported satisfaction levels also introduces an element of subjectivity, as responses may be influenced by emotional states, recall bias, or personal experiences, which could distort the actual satisfaction levels. Furthermore, the inclusion of patients with longer ICU stays may introduce a bias, as prolonged stays could be associated with lower family satisfaction due to the increased stress and potential negative outcomes over time. This factor should be considered when interpreting the satisfaction levels reported by families in this study. These limitations necessitate caution in interpreting and applying our findings beyond the studied context.

## 5. Conclusion

This study identified generally moderate to high levels of family satisfaction within the ICU, revealing notable gender-related differences in satisfaction. The key factors enhancing satisfaction included effective communication and active family participation in decision-making. These findings suggest the need for ICUs to implement regular assessments of family satisfaction to inform targeted quality improvement initiatives. Future research should explore the underlying causes of satisfaction variations and develop interventions aimed at refining the ICU experience for both patients and their families.

## Figures and Tables

**Table 1:** Demographic and clinical characteristics of intensive care patients.

	Category	Count (%)	Overall mean ± SD	*p* value
Gender	Female	176 (70.97%)	3.61 ± 1.11	0.007^a^
	Male	72 (29.03%)	4.24 ± 1.20	

Relationship with patient	Offspring	84 (33.87%)	3.65 ± 0.97	0.594^b^
	Other family member	32 (12.90%)	3.77 ± 1.45	
	Parents	104 (41.94%)	3.90 ± 1.33	
	Siblings	16 (6.45%)	3.98 ± 0.97	
	Spouse	12 (4.84%)	3.75 ± 0.80	

Type of intensive care	Medical ICUs	124 (50.00%)	3.85 ± 1.22	0.636^b^
	Pediatric ICUs	60 (24.19%)	3.58 ± 1.27	
	Surgical ICUs	64 (25.81%)	3.89 ± 0.99	

^a^Mann‒Whitney *U* test (U) and ^b^Kruskal‒Wallis test.

**Table 2:** Patient satisfaction survey results in the intensive care unit.

Subscales	Items	Dissatisfied	Not Sure	Satisfied	Mean ± SD
Assurance	Waiting time for results of tests and X-rays	40 (16.13%)	44 (17.74%)	164 (66.13%)	3.73 ± 1.28
	Peace of mind in knowing my family member's nurse(s)	32 (12.90%)	44 (17.74%)	172 (69.35%)	3.82 ± 1.18
	Promptness of staff in responding to alarms and requests for assistance	52 (20.96%)	40 (16.13%)	156 (62.91%)	3.71 ± 1.3
	Noise in the critical care unit	24 (9.68%)	48 (19.35%)	176 (70.97%)	3.92 ± 1.13
	Sharing in decisions regarding my family member's recovery	24 (9.68%)	52 (20.97%)	172 (69.35%)	3.95 ± 1.11

Proximity	Ability to share in the care of my family member	40 (16.13%)	36 (14.52%)	172 (69.35%)	3.76 ± 1.28
	Privacy provided for me and my family member during our visits	36 (14.52%)	44 (17.74%)	168 (67.74%)	3.82 ± 1.26
	Flexibility of visiting hours	52 (20.97%)	48 (19.35%)	148 (59.68%)	3.58 ± 1.31

Information	Availability of the doctor to speak with me on a regular basis	44 (17.74%)	28 (11.29%)	176 (70.97%)	3.84 ± 1.3
	Clear explanation of tests, procedures, and treatments	40 (16.13%)	32 (12.9%)	176 (70.97%)	3.82 ± 1.18
	Clear answers to my questions	36 (14.52%)	44 (17.74%)	168 (67.74%)	3.84 ± 1.27
	Sharing in decisions regarding my family member's care on a regular basis	32 (12.90%)	44 (17.74%)	172 (69.36%)	3.82 ± 1.26

Support	Honesty of the staff about my family member's condition	28 (11.29%)	40 (16.13%)	180 (72.58%)	3.9 ± 1.17
	Support and encouragement given to me during my family member's stay in the critical care unit	36 (14.52%)	48 (19.35%)	164 (66.13%)	3.76 ± 1.24
	Quality of care given to my family member	48 (19.36%)	32 (12.9%)	168 (67.75%)	3.71 ± 1.38
	Nurses' availability to speak with me every day about my family member's care	40 (16.13%)	36 (14.52%)	172 (69.35%)	3.82 ± 1.31
	Sensitivity of doctor(s) to my family member's needs	36 (14.52%)	40 (16.13%)	172 (69.35%)	3.85 ± 1.27
	Preparation for my family member's transfer from critical care	52 (20.97%)	44 (17.74%)	152 (61.29%)	3.63 ± 1.33

Comfort	Cleanliness and appearance of the waiting room	52 (20.97%)	28 (11.29%)	168 (67.75%)	3.84 ± 1.26
	Peacefulness of the waiting room	36 (14.52%)	44 (17.74%)	168 (67.75%)	3.79 ± 1.28

**Table 3:** Mean scores and standard deviations for each subscale and the overall family satisfaction score.

Subscale	Mean ± SD
Assurance	3.82 ± 1.20
Information	3.83 ± 1.25
Comfort	3.81 ± 1.27
Proximity	3.72 ± 1.28
Support	3.78 ± 1.28
Overall family satisfaction	3.79 ± 1.26

## Data Availability

The data that support the findings of this study are available from the corresponding author upon reasonable request.
